# Phase-Modulated Waveform Design for Extended Target Detection in the Presence of Clutter

**DOI:** 10.3390/s110707162

**Published:** 2011-07-12

**Authors:** Xuhua Gong, Huadong Meng, Yimin Wei, Xiqin Wang

**Affiliations:** Department of Electronic Engineering, Tsinghua University, Beijing 100084, China; E-Mails: gongxh07@mails.tsinghua.edu.cn (X.G.); weiym@mails.tsinghua.edu.cn (Y.M.); wangxq_ee@tsinghua.edu.cn (X.W.)

**Keywords:** clutter, constant modulus waveform, radar waveform design, target detection

## Abstract

The problem to be addressed in this paper is a phase-modulated waveform design for the detection of extended targets contaminated by signal-dependent noise (clutter) and additive noise in practical radar systems. An optimal waveform design method that leads to the energy spectral density (ESD) of signal under the maximum signal-to-clutter-and-noise ratio (SCNR) criterion is introduced first. In order to make full use of the transmission power, a novel phase-iterative algorithm is then proposed for designing the phase-modulated waveform with a constant envelope, whose ESD matches the optimal one. This method is proven to be able to achieve a small SCNR loss by minimizing the mean-square spectral distance between the optimal waveform and the designed waveform. The results of extensive simulations demonstrate that our approach provides less than 1 dB SCNR loss when the signal duration is greater than 1 μs, and outperforms the stationary phase method and other phase-modulated waveform design methods.

## 1. Introduction

The problem of transmit waveform design for optimal detection, given some knowledge of the targets and the environment, has been a problem of long-standing interest. In actual radar systems, it is necessary to select the operating band, transmit waveform modulation, and receiver processing strategy judiciously in order to maximize the probability of detecting the presence of a target while maintaining a prescribed rate of false alarms [1]. Additionally, the fact that the received clutter characteristics are dependent on the transmit signal greatly complicates the optimal signal design. Furthermore, as the resolution of a radar system improves, the assumption of a point-target, which has a flat response (and linear phase) across the instantaneous operating band of the radar, does not hold because the spatial area occupied by the observed target exceeds one resolution cell. In such cases, the extended target model is proposed as a means of accurately representing the behavior of observed targets. In this paper, we consider a phase-modulated waveform design for the detection of extended target surrounded by clutter and additive noise in practical radar systems.

Much earlier work has been presented on the techniques of waveform design for detecting targets [2–4] and on waveform design for imaging in the presence of clutter [5]. Bell introduced two different paradigms for waveform design: one that used the signal-to-noise-ratio (SNR) criterion and one that used the mutual information (MI) criterion [6]. Waveform designs created using both criteria were used to improve the performance of a closed-loop radar system applied to target recognition [7]. Additionally, the optimum transmit-receiver design problem in the presence of clutter was investigated in [8]. Based on maximizing the output signal-to-interference-plus-noise ratio (SINR), an iterative solution for the transmit waveform and its companion receiver for extended target detection was proposed, which is neither guaranteed to converge nor to produce the optimal signal. In [9], the analytic solution of optimal transmit waveform for Gaussian point target detection was obtained based on the likelihood ratio test (LRT). The technique of waveform design was heuristically extended to tackle the multistatic radar detection problems in [10]. The relationship between two measurement metrics, which are minimum mean squared error (MMSE) in statistical signal estimation theory and MI in information theory, was first discussed in [11]. The use of MMSE and MI criterions in designing waveforms was extended for multiple-input multiple-output (MIMO) parameter estimation and target identification in [12,13]. The matched radar transmit waveform design based on the MI criterion for multiple extended targets was presented in [14]. In [15], the design of matched waveforms based on maximization of both SNR and MI was treated. Though these results show the benefits of transmit waveform design for target detection, estimation and recognition, the assumption of an arbitrary waveform used in previous literatures [6–15] is inappropriate for a practical radar system because it is extremely difficult to implement. Patton uses hardware considerations to argue that constraints on the maximum waveform modulus will generally supersede the commonly found total energy constraint [16]. The maximum waveform modulus constraint is more suitable for practical radar systems, and a constant modulus waveform can fully exploit the power of the transmitter. The constant modulus constraint was previously discussed and adopted in the optimal waveform design for improving target detection [17]. The phase-coded waveform design for target detection and recognition was investigated in [18,19]. In [20], a method based on phase-modulated signal was proposed to exploit the transmit capability, whose effect would be deteriorated in the heavy clutter. The stationary-phase method can commonly be used to design or synthesize a nonlinear phase-modulated signal of a large time-bandwidth product [21]. However, it is difficult to obtain the designed signal in accordance with the arbitrary auto-correlation function [22]. In [23], a method based on the linear least squares estimate was arose that was not necessarily optimal. In [24], the design of unimodular sequences with good autocorrelation properties was solved by minimizing the integrated sidelobe level (ISL) of sequences.

In this paper, we only consider zero Doppler targets that represent the worst-case scenario, and describe an approach that yields the applicable phase-modulated waveform for extended target detection in the presence of clutter. The scattering behavior of extended target that can be described by the impulse response function [6,8,9], which is referred to as the high resolution range profile (HRRP) in radar automatic target recognition (ATR) problem [25], can be estimated and known as prior information. The clutter returns are assumed to be the output of a linear time invariant filter with a stochastic impulse response driven at the input by the transmit waveform. From the standpoint of hardware realization, we use the phase-modulated waveform because it can make full use of the transmit power in the pulse duration under the maximum modulus constraint.

The main contribution of this paper is the method of appropriate phase-modulated waveform design for extended target detection based on the optimal waveform derived from the maximum SCNR constraint. Considering the general requirements of waveform on finite duration and energy [6], an analytical solution of the optimal signal ESD that can maximize the output SCNR is obtained. Therefore, the phase-modulated waveform design method is proposed according to minimizing the mean-square spectral distance between the optimal ESD and the ESD of the realized waveform. We also contribute by proving that the minimum mean-square spectral distance can lead to the small SCNR loss.

The paper is organized as follows. In Section 2, we present the system model and define the problem statement. In Section 3, considering the finite waveform energy and duration time, we introduce an optimal waveform design method for extended target detection according to SCNR criteria. In Section 4, we introduce a phase-iterative algorithm for designing the appropriate phase-modulated waveform. In Section 5, we present the performance results and discuss the proposed waveform design algorithms. Our conclusions are given in Section 6.

## 2. Problem Formulation and System Model

**Figure 1.**
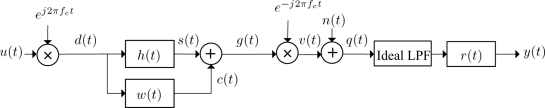
Block diagram of radar system model.

The block diagram in Figure 1 illustrates our simplified model of a practical radar system and its essential features. In the model, *u*(*t*) is a complex-valued finite duration baseband transmit waveform for −*T/*2 ≤ *t* ≤ *T/*2, where *T* is the signal duration time. The scattering of target and clutter are viewed as linear processes that are modelled by the system impulse response *h*(*t*) and *w*(*t*) respectively, here *h*(*t*) is the impulse response of the extended target and is assumed to be deterministic and integrable and *w*(*t*) is a zero-mean complex wide sense stationary (WSS) Gaussian random process with known power spectral density (PSD) *P_ww_*(*f*). The transmit signal *u*(*t*) is modulated to the transmission carrier frequency *f_c_* and passes through the target channel *h*(*t*) and clutter random channel *w*(*t*), the return echo is demodulated to baseband signal *v*(*t*) and then passes the ideal low-pass filter (LPF) with passband [−*B*/2, *B*/2]. In addition, *n*(*t*) is the zero-mean additive WSS Gaussian noise with known PSD *P_nn_*(*f*) and is supposed to be independent of *u*(*t*), *h*(*t*) and *w*(*t*). Because the impulse response *h*(*t*) and *w*(*t*) are assumed to be independent in practical applications, the target echo *s*(*t*) and the clutter returns *c*(*t*) are also independent when the transmitted signal *u*(*t*) is deterministic and accurately known.

From the system model in Figure 1, we obtain that the output signal *y*(*t*) is:

(1) $$\begin{array}{ll} y(t) & =\mathrm{LPF}[q(t)]*r(t)\\ & =\mathrm{LPF}[v(t)]*r(t)+\mathrm{LPF}[n(t)]*r(t)\\ & =\mathrm{LPF}[s(t)e^{-j2\pi f_{c}t}]*r(t)+\mathrm{LPF}[c(t)e^{-j2\pi f_{c}t}]*r(t)+\mathrm{LPF}[n(t)]*r(t)\\ & ≜y_{s}(t)+y_{c}(t)+y_{n}(t) \end{array}$$

where *LPF*[·] represents the ideal low-pass filter with passband [−*B*/2, *B*/2] and *y_s_*(*t*), *y_c_*(*t*) and *y_n_*(*t*) are the target echo component, clutter returns and additive noise, respectively. Additionally, the transmit waveform *u*(*t*) must satisfy the finite duration and energy constraints [6] as follows:

(2) $$u(t)=0,\;\;t\notin \left[-\frac{T}{2},\frac{T}{2}\right]$$

(3) $$\int_{-B/2}^{B/2}|U(f){|}^{2}df=E$$

where *E* is the total transmit energy and *U*(*f*) is the Fourier transform of *u*(*t*) respectively.

## 3. SCNR-Based Waveform Design in Clutter

Referring to the previous modeling assumptions and formulations, the receiver output SCNR at time *t*_0_ is defined as:

(4) $${\text{SCNR}}_{{\text{t}}_{0}}=\frac{E(|y_{s}(t_{0}){|}^{2})}{E(|y_{c}(t_{0}){|}^{2})+E(|y_{n}(t_{0}){|}^{2})}$$

Then, our objective is to jointly optimize the transmit waveform *u*(*t*) and the receiver-filter *r*(*t*) for maximizing SCNR_t_0__ defined in (4). Let *S*(*f*), *C*(*f*) and *V*(*f*) be the Fourier transforms of *s*(*t*), *c*(*t*) and *v*(*t*) respectively; the energy of target echo, clutter and noise are:

(5) $$E(|y_{s}(t_{0}){|}^{2})=\left|\int_{-B/2}^{B/2}U(f)H(f+f_{c})R(f)e^{j2\pi ft_{0}}df\right|$$

(6) $$E(|y_{c}(t_{0}){|}^{2})=\int_{B/2}^{B/2}|R(f){|}^{2}P_{ww}(f+f_{c})|U(f){|}^{2}df$$

(7) $$E(|y_{n}(t_{0}){|}^{2})=\int_{B/2}^{B/2}|R(f){|}^{2}P_{nn}(f)df$$

where *R*(*f*) is the Fourier transform of the receiver-filter impulse response *r*(*t*) and SCNR_t_0__ becomes:

(8) $$\begin{array}{l} {\text{SCNR}}_{{\text{t}}_{0}}\;=\frac{{\left|\int_{-B/2}^{B/2}U(f)H(f+f_{c})R(f)e^{j2\pi ft_{0}}df\right|}^{2}}{\int_{-B/2}^{B/2}|R(f){|}^{2}L(f)df}\\ \;\;\;\;\;\;\;\;\;\;\;\;\;\;\;\;\;=\frac{{\left|\int_{-B/2}^{B/2}R(f)\sqrt{L(f)}\frac{U(f)H(f+f_{c})e^{j2\pi ft_{0}}}{\sqrt{L(f)}}df\right|}^{2}}{\int_{-B/2}^{B/2}|R(f){|}^{2}L(f)df} \end{array}$$

where *L*(*f*) = *P_nn_*(*f*) + *P_ww_*(*f* + *f_c_*)|*U(f*)|^2^. With the application of the Cauchy–Schwarz inequality to Equation (8), we have:

(9) $${\text{SCNR}}_{{\text{t}}_{0}}\leq {\int_{-B/2}^{B/2}\left|\frac{U(f)H(f+fc)}{\sqrt{L(f)}}\right|}^{2}df$$

with equality holds if and only if:

(10) $$R(f)=K\frac{U*(f)H*(f+f_{c})}{L(f)}e^{-j2\pi ft_{0}}$$

where *K* is a complex constant. In Equation (10), the optimal receiver-filter is related to the target impulse response and clutter returns, in contrast to the matched-filter in a traditional radar system. A similar result can be found in [6] without the presence of clutter. The SCNR to be maximized is now given by:

(11) $$\text{SCNR}=\int_{-B/2}^{B/2}\frac{|U(f){|}^{2}|H(f+f_{c}){|}^{2}}{P_{nn}(f)+P_{ww}(f+f_{c})|U(f){|}^{2}}df$$

Considering the imposed constraints of finite energy and duration time on the transmit waveform given in Equations (2) and (3), the Lagrangian multiplier technique can be applied to obtain the maximization of SCNR in Equation (11). The objective function is constituted as:

(12) $$\begin{array}{l} I(|U(f){|}^{2})=\int_{-B/2}^{B/2}\frac{|U(f){|}^{2}|H(f+f_{c}){|}^{2}}{P_{nn}(f)+P_{ww}(f+f_{c})|U(f){|}^{2}}df-\lambda \left(\int_{-B/2}^{B/2}|U(f){|}^{2}df-E\right)\\ \;\;\;\;\;\;\;\;\;\;\;\;\;\;\;\;\;\;\;\;\;\;\;\;\;=\int_{-B/2}^{B/2}\left(\frac{|U(f){|}^{2}|H(f+f_{c}){|}^{2}}{P_{nn}(f)+P_{ww}(f+f_{c})|U(f){|}^{2}}-\lambda |U(f){|}^{2}\right)df+\lambda E \end{array}$$

where λ is a constant. Therefore, the maximization of *I*(|*U*(*f*)|^2^) can be reduced to maximizing *Q*(|*U*(*f*)|^2^), which is defined by:

(13) $$Q\left(|U(f){|}^{2}\right)=\frac{|U(f){|}^{2}|H(f+f_{c}){|}^{2}}{P_{nn}(f)+P_{ww}(f+f_{c})|U(f){|}^{2}}-\lambda |U(f){|}^{2}$$

Let the first derivative of Equation (13) with respect to the signal ESD |*U*(*f*)|^2^ equal zero to obtain that:

(14) $$\frac{P_{nn}(f)|H(f+f_{c}){|}^{2}}{{\left(P_{nn}(f)+P_{ww}(f+f_{c})|U(f){|}^{2}\right)}^{2}}-\lambda =0$$

Considering the nonnegativity of ESD, the optimal ESD of transmit waveform that can maximize output SCNR is that:

(15) $$\begin{array}{l} \varepsilon_{opt}(f)=|U_{\mathrm{opt}}(f){|}^{2}\\ \;\;\;\;\;\;\;\;\;\;\;\;\;\;\;\;\;=\text{max}\left(\frac{\sqrt{P_{nn}(f)|H(f+f_{c}){|}^{2}/\lambda }-P_{nn}(f)}{P_{ww}(f+f_{c})},0\right),-\frac{B}{2}\leq f\leq \frac{B}{2} \end{array}$$

where λ is a constant that can be numerically found from the energy constraint in Equation (3), it is a one-dimensional search problem. Therefore, the maximum of output SCNR is given by:

(16) $${\text{SCNR}}_{\text{max}}=\int_{-B/2}^{B/2}\frac{{ɛ}_{opt}(f)|H(f+f_{c}){|}^{2}}{P_{nn}(f)+P_{ww}(f+f_{c}){ɛ}_{opt}(f)}df$$

From the above analysis, the optimal ESD of complex-valued transmit waveform that maximizes the output SCNR at the receiver can be illustrated by the “water-filling” model [26]. This similar result is also described in [15,27].

## 4. Phase-Modulated Waveform Design

So far in this paper, an analytical solution for extended target detection is derived from SCNR constraints; under this condition, the time-domain signal can be synthesized by using finite impulse response filter design techniques and Durbin’s method as described in [28]. However, the synthetic signal that contains amplitude modulation cannot fully exploit the power of a transmitter in the practical radar system, the constraint of maximum waveform modulus is more suitable for the practical implementation [16]. Based on these considerations, a phase-iterative algorithm is proposed for use in designing an appropriate phase-modulated waveform that can approximate the optimal ESD in the mean-square sense.

We now consider the problem of finding waveforms with constant envelope that approximate the optimal ESD. We assume that *u_pm_*(*t*) is a complex-valued phase-modulated waveform given by:

(17) $$u_{\mathrm{pm}}(t)=ce^{j\phi (t)},\;\;\;\;t\in \left[-\frac{T}{2},\frac{T}{2}\right]$$

where *c* is the constant-valued modulus dependent on the transmitter power. The ESD of signal *u_pm_*(*t*) will then be:

(18) $${ɛ}_{\mathrm{pm}}(f)=|U_{\mathrm{pm}}(f){|}^{2}={\left|\int_{-T/2}^{T/2}u_{\mathrm{pm}}(t)e^{-j2\pi ft}dt\right|}^{2}$$

where |·| is the absolute value operator.

Our problem can be further stated as “find the appropriate signal *u_pm_*(*t*) that can make the output SCNR difference between the optimal transmit waveform and phase-modulated waveform as small as possible”, which is defined by:

(19) $$\Delta \text{SCNR}={\text{SCNR}}_{\text{max}}-{\text{SCNR}}_{\text{pm}}$$

where SCNR_max_ is the maximal value of output SCNR given by Equation (16) and SCNR_pm_ is the value of output SCNR under the phase-modulated signal *u_pm_*(*t*). However, it is difficult to obtain straightforward minimization of ΔSCNR by designing *u_pm_*(*t*). From the definition of SCNR in Equation (11), we can make the ESD of *u_pm_*(*t*) given in Equation (18) best approximate the optimal transmit ESD given in Equation (15) by finding the signal phase term *ϕ*(*t*) and using:

(20) $$\begin{array}{l} G(\phi (t))=\int_{-B/2}^{B/2}{\left({ɛ}_{\mathrm{pm}}(f)-{ɛ}_{\mathrm{opt}}(f)\right)}^{2}df\\ \;\;\;\;\;\;\;\;\;\;\;\;\;\;\;\;\;\;={\int_{-B/2}^{B/2}\left(c^{2}{\left|\int_{-T/2}^{T/2}e^{j(\phi (t)-2\pi ft)}dt\right|}^{2}-{ɛ}_{\mathrm{opt}}(f)\right)}^{2}df \end{array}$$

to describe the ESD difference between the phase-modulated waveform and the optimal transmit waveform in the mean square sense.

Our next objective is to minimize the ESD difference *G*(*ϕ*(*t*)) within the passband [–*B*/2, *B*/2]. In Appendix A, we derive that the approximation in Equation (20) can promise a small output SCNR difference, which can be described by:

(21) $$\Delta \mathrm{SCNR}\leq \tilde{\text{K}}\sqrt{G(\phi (t))}$$

where K̃ is a constant related to the PSD of additive noise *P_nn_*(*f*) and the spectrum of extended target impulse response *H*(*f* + *f_c_*). Because the ESD difference *G*(*ϕ*(*t*)) can converge to zero when the signal duration time *T* is infinite [29], the SCNR_pm_ will equal the SCNR_max_ in Equation (21). Therefore, the minimization of the ESD difference *G*(*ϕ*(*t*)) can make SCNR_pm_ approximate the upper bound SCNR_max_ as closely as possible.

Because most of the radar systems are digitized, and for the convenience of simulation, we use a discrete-time formulation to replace the representation of signal in Equation (20). We assume that *T_s_* is the sampling interval, then the signal in discrete time is defined as:

(22) $$u=c{\left[e^{j\phi_{0}},\;\;\;\;e^{j\phi_{1}},\;\;\;\;\;\cdots \;,\;\;\;\;e^{j\phi_{N-1}},\right]}^{T}$$

and the phase vector is denoted as:

(23) $$\phi ={\left[\phi_{0},\;\;\phi_{1},\;\;\;\cdots ,\;\;\phi_{N-1}\right]}^{T}$$

where [·]*^T^* is the transposed operator, *N* = *T*/*T_s_*. The function defined in (20) can be obtained discretely using the Discrete Fourier Transform of u, which is:

(24) $$G(\phi )=F_{s}\sum_{m=0}^{M-1}{\left({\left|cT_{s}\sum_{n=0}^{N-1}e^{j(\phi_{n}-2\pi f_{m}nT_{s})}\right|}^{2}-{ɛ}_{opt}(f_{m})\right)}^{2}$$

where *M* = *B*/*F_s_*, *f_m_* = −*B*/2 + *mF_s_*, $c=\sqrt{E/T}$, *F_s_* is the sampling interval in the frequency domain and *E* is the transmit energy. Due to the nonlinearity, it is difficult to find an analytical solution that minimizes Equation (24). To solve this problem, an iterative approach in which the calculation in each step is analytical is proposed.

Because it is difficult to conjointly solve the phase vector *ϕ*, we can optimize the elements of phase vector *ϕ* one by one. This method of updating *ϕ* can also keep the ESD difference *G*(*ϕ*) decreasing monotonically with each iterative step. In Appendix B, we find that the appropriate phase element *ϕ_k_* that satisfies *∂G*(*ϕ*)/*∂ϕ_k_* = 0 and $\partial^{2}G(\phi )/\partial \phi_{k}^{2}>0$ can minimize *G*(*ϕ*), where *∂G*(*ϕ*)/*∂ϕ_k_* is the first-order partial derivative of *G*(*ϕ*) with respect to phase *ϕ_k_*, which indicates the phase of *u_pm_*(*t*) at the *k_th_* sampling time, and $\partial^{2}G(\phi )/\partial \phi_{k}^{2}$ is the second-order partial derivative. Therefore, the elements of phase vector *ϕ* from *ϕ*_0_ to *ϕ_N–_*_1_ can be sequentially adjusted.

The phase-iterative algorithm for phase-modulated waveform design can be summarized as follows:

1. Initialize the phase vector *ϕ*, e.g., *ϕ*^(0)^ = [0, ⋯, 0]*^T^*.
2. Let the initial number be *p* = 0 and calculate the ESD difference *G*^(0)^.
3. *p* = *p* + 1.
4. Set *k* from 0 to *N* – 1 and find the solution $\phi_{k}^{\left(p\right)}$ of the equation $\partial G(\phi )/\partial \phi_{k}^{\left(p\right)}=0$ by using Appendix B as a guide, which satisfies $\partial^{2}G(\phi )/\partial {(\phi_{k}^{\left(p\right)})}^{2}>0$.
5. The updated signal is **u**^(*p*)^ = *ce*^*jϕ*^(*p*)^^, *G*^(*p*)^ = *G*(*ϕ*^(*p*)^) and *δ* = *G*^(*p*)^ – *G*^(*p*−1)^.
6. If *δ* > *D*, where *D* is a predefined threshold, go to step (3); otherwise, **u**^(*p*)^ is the solution.

Since *G* (*ϕ*) decreases monotonically with each iterative step, the phase-modulated waveform ESD *ɛ_pm_*(*f*) can approximate the optimal transmit ESD *ɛ_opt_*(*f*) as much as possible in the mean-square sense.

## 5. Simulations and Discussions

In this section, numerical results are presented and some discussions are conducted to evaluate the performance of the optimal waveform design for extended target detection and the proposed phase-iterative algorithm for the phase-modulated waveform design.

### 5.1. Optimal ESD of Transmit Waveform Design

#### 5.1.1. A Numerical Example

**Figure 2.**
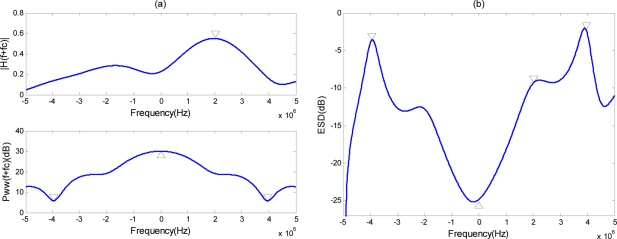
**(a)** Amplitude spectrum of an extended target impulse response and clutter random channel PSD; **(b)** Optimal ESD of the transmit waveform.

We now consider the following scenario. The carrier frequency *f_c_* is 10 *GHz*, the bandwidth *B* is 10 *MHz*, the energy of transmit signal *E* is 10^6^ *joules*, and the additive noise is white with PSD *P_nn_*(*f*) = 1 for *f* ∈ [−*B*/2, *B*/2]. A target with multiple reflection centers according to the multiple reflection model [30] is adopted here, the impulse response *h*(*t*) of which can be denoted as $\sum_{i=1}^{K}h_{i}\delta (t-t_{i})$, the number of reflection centers *K* is 15. The reflection coefficients *h_i_* are assumed to follow a zero-mean Gaussian distribution with unit variance. The different delays *t_i_* are the independent samples following a uniform distribution between 0 and 0.33 μs, which means that the range span of the target is 50 *m*. The upper subfigure of Figure 2(a) shows the amplitude spectrum of an extended target; the lower subfigure shows the PSD of the clutter random channel. The optimal ESD of transmit waveform is shown in Figure 2(b). As expected, the optimal transmit ESD for extended target detection places as much energy as possible into the mode of the extended target that gives the largest response when weighted with the clutter. This demonstrates that the waveform design for optimal target detection de-emphasizes the frequency bands where clutter is significant and emphasizes the frequency bands where the target response is significant.

#### 5.1.2. Point Target Assumption

Under this assumption, the impulse response of a point target can be represented by a delta function; therefore, the amplitude spectrum of the target impulse response will be a specified value *A* for the passband [−*B*/2, *B*/2]. The optimal transmit ESD for extended target detection in Equation (15) is in accordance with that for point target detection, and the optimal ESD of signal is:

(25) $$|U(f){|}^{2}=\text{max}\left(\frac{\sqrt{A^{2}P_{nn}(f)/\lambda }-P_{nn}(f)}{P_{ww}(f+f_{c})},0\right)$$

here *f* ∈ [−*B*/2, *B*/2]. The additive noise is assumed to be white with PSD *P_nn_*(*f*) = *N*_0_, where *N*_0_ is a constant. To simplify the discussion, the value of *A* is assumed to be $\sqrt{N_{0}}$, so that $|H(f+f_{c}){|}^{2}/P_{nn}(f)=1$. Then, we assume that the transmit energy *E* is large enough so as to make λ ≤ 1. The optimal transmit ESD |*U*(*f*)|^2^ in Equation (25) will be:

(26) $$|U(f){|}^{2}=\frac{N_{0}(\sqrt{1/\lambda }-1)}{P_{ww}(f+f_{c})}$$

For the transmit energy constraint in Equation (3) to hold, we have:

(27) $$|U(f){|}^{2}=\frac{E\int_{-B/2}^{B/2}P_{ww}(f+f_{c})df}{P_{ww}(f+f_{c})}$$

As we can see from Equation (27), |*U*(*f*)|^2^ = *ξ*/*P_ww_*(*f* + *f_c_*), where *ξ* is a constant. The PSD of clutter returns *c*(*t*) will be *P_cc_*(*f* + *f_c_*) = |*U*(*f*)|^2^ *P_ww_*(*f* + *f_c_*) = *ξ*, it means the optimal signal can whiten the clutter. This result is the same as Kay’s given in [9].

### 5.2. Phase-modulated Waveform Design

#### 5.2.1. A Numerical Example and Statistical Results

We now consider an example to illustrate the proposed method for designing the phase-modulated waveform and examine the characteristics of the designed waveform. The signal duration time *T* is 3 μs, and the sampling interval *T_s_* is 1/(20*B*). The optimal transmit ESD shown in Figure 2(b) is employed as the approximated ESD. For convenience, the optimal transmit waveform is normalized to ‖*U_opt_*(*f*)‖ = 1, here ‖·‖ is the 2-norm operator.

**Figure 3.**
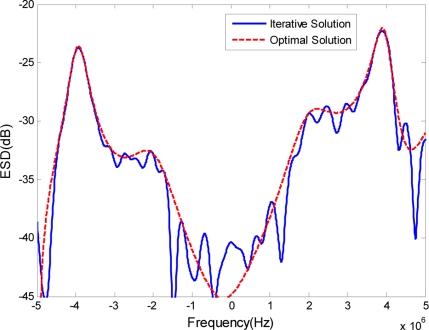
ESD of the optimal transmit waveform and the phase-modulated waveform.

**Figure 4.**
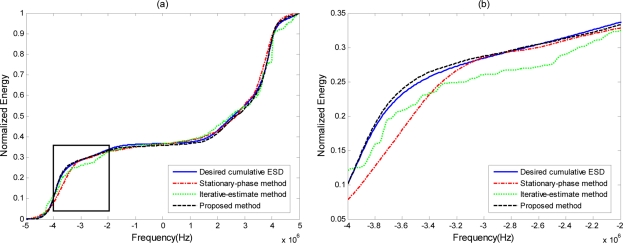
Comparison of the normalized cumulative ESD of signals synthesized using different methods for **(a)** the full passband and **(b)** the zoomed version of the section highlighted in a box.

The optimal transmit ESD when the cut-off threshold *D* is 10^−5^ is shown in Figure 3 as a short dashed curve, along with the ESD of the phase-modulated waveform, which is shown as a solid curve. It is expected that the ESD of the phase-modulated waveform designed by our approach will appear very similar to the optimal transmit ESD. The output SCNR under the phase-modulated signal SCNR_pm_ is 28.35 dB, while the maximal receiver output SCNR under the optimal transmit waveform SCNR_max_ is 28.83 dB. The level of similarity between the ESD of the designed phase-modulated waveform using the proposed method and the optimal ESD can be visualized better by the normalized cumulative ESDs in Figure 4. At the same time, it can be compared against the cumulative ESD of the signal synthesized using the stationary-phase method [21] and the iterative-estimate method [23]. Normalized cumulative ESD is defined as:

(28) $$\hat{ɛ}\left(f_{k}\right)=\frac{\sum_{m=0}^{k}ɛ(f_{m})}{\sum_{m=0}^{M-1}ɛ(f_{m})},\;\;\;\;k=0,\;\;\;...,\;M-1$$

**Figure 5.**
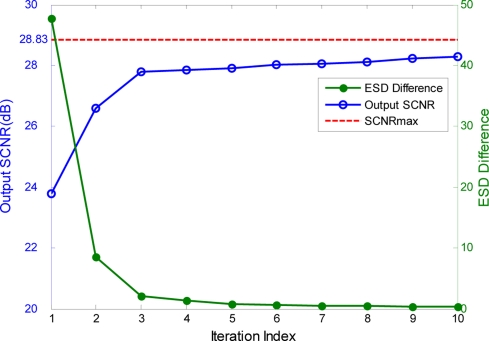
Output SCNR and ESD difference *versus* number of iterations.

The zoomed version of the section highlighted in a box in Figure 4(a) is shown in Figure 4(b). As we can see, the stationary—phase and iterative-estimate methods deviate from the desired cumulative ESD around the edges, while the proposed method follows the desired cumulative ESD very closely. This means that the proposed method outperforms the stationary-phase and iterative-estimate methods when the desired ESD has many sudden fluctuations. Figure 5 shows the variation of the output SCNR (the left longitudinal axis of the coordinates) and the ESD difference *G*(*ϕ*) (the right longitudinal axis of coordinates) over 10 iterative steps using the proposed method. We can see that with decreasing *G*(*ϕ*) the corresponding output SCNR under the designed phase-modulated signal, shown by the dashed curve, converges to the maximal SCNR and that it achieves 0.53 dB difference at the 10th iteration. From the above results, it can be concluded that the phase-modulated waveform designed by our proposed phase-iterative algorithm can achieve satisfactory output SCNR approximation to the maximal output SCNR.

**Figure 6.**
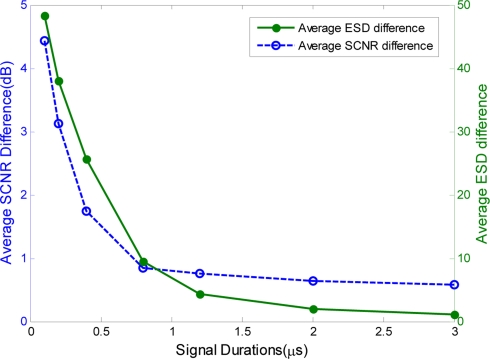
Average ESD difference and SCNR difference.

In additional, we performed Monte Carlo simulations to characterize the performance of our proposed phase-iterative algorithm. Figure 6 shows the results of a Monte Carlo simulation with 1000 target samples generated according to the multiple reflection model [30] and 1,000 samples of the clutter power spectrum [31]. The average ESD difference and SCNR difference are used to evaluate the performance of the proposed method. As shown in Figure 6, the average SCNR difference decreases and converges to zero with increased signal duration because the monotonically-decreasing ESD difference *G*(*ϕ*) is much smaller with the longer signal duration.

#### 5.2.2. Initialization of Phase-iterative Algorithm

**Figure 7.**
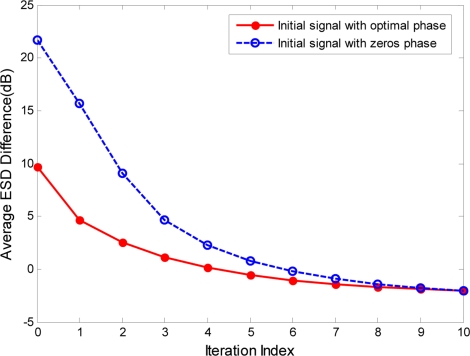
Average ESD difference and SCNR difference.

The initialization of the phase vector *ϕ* in the proposed phase-iterative algorithm is a considerable problem that can affect the convergence of the iterative solutions. In our approach, the phase vector *ϕ* of the phase-modulated signal *u_pm_*(*t*) is always initialized to zeros and will be adjusted within the iterations to reduce the ESD difference *G*(*ϕ*). Because the phase term of *u_pm_*(*t*) contains the spectrum characteristics of *U_pm_*(*f*), the phase vector *ϕ* can also be initialized to the angle of optimal signal *u_opt_*(*t*), which is the inverse Fourier transform of the optimal transmit spectrum *U_opt_*(*f*). In this way, more energy of *U_pm_*(*f*) can be concentrated at a frequency band in which the optimal ESD *ɛ_opt_*(*f*) is much larger. However, from the definition of ESD, the phase term of *u_opt_*(*t*) can not be directly obtained from the optimal ESD *ɛ_opt_*(*f*). We therefore assume that the phase of the optimal spectrum *U_opt_*(*f*) is represented by an independent sampling of a uniform distribution on [−π, π]. Figure 7 shows the results of a Monte Carlo simulation with 1,000 target samples and clutter power spectrum samples when the signal duration *T* is assumed to be 2 μs. As seen in Figure 7, the phase initialization of signal *u_pm_*(*t*) by the optimal phase, which is the angle of the optimal signal *u_opt_*(*t*), can achieve a better ESD approximation with fewer iterations.

## 6. Conclusions

The problem of detecting targets in clutter has been the subject of much research and has led to the development of clutter rejecting waveforms. In this paper, we perform the phase-modulated waveform design for approximating to the optimum performance of the extended target detection. A simple theoretical solution of the optimal ESD of complex-valued transmit waveform is obtained. Based on this, we present a novel phase-iterative method for designing the phase-modulated waveform, aiming to minimize the mean-square spectral distance between the optimal waveform and the designed waveform, which can promise the approximation of the output SCNR to the maximum SCNR. The numerical simulations show that the advantage of the proposed phase-iterative waveform design technique is substantial, and that our approach outperforms the stationary phase method and other phase-modulated waveform design methods.

## Appendix A

As described in Section 4, we assume that *ɛ_opt_*(*f*) is the optimal ESD of the transmit waveform *u_opt_*(*t*) and that *ɛ_pm_*(*f*) is the ESD of the phase-modulated waveform *u_pm_*(*t*). The objective function to be minimized is:

(29) $$G(\phi (t))=\int_{-B/2}^{B/2}{\left({ɛ}_{\mathrm{pm}}(f)-{ɛ}_{opt}(f)\right)}^{2}df$$

From the results in Section 3, the maximum receiver output SCNR is given by:

(30) $${\text{SCNR}}_{\text{max}}=\int_{-B/2}^{B/2}\frac{{ɛ}_{opt}(f)|H(f+f_{c}){|}^{2}}{P_{nn}(f)+P_{ww}(f+f_{c}){ɛ}_{opt}(f)}df$$

Therefore, when the transmit waveform is a phase-modulated signal *u_pm_*(*t*), the output SCNR is calculated by:

(31) $${\text{SCNR}}_{\text{pm}}=\int_{-B/2}^{B/2}\frac{{ɛ}_{\mathrm{pm}}(f)|H(f+f_{c}){|}^{2}}{P_{nn}(f)+P_{ww}(f+f_{c}){ɛ}_{\mathrm{pm}}(f)}df$$

For simplicity, let *P_ww_*(*f* + *f_c_*) = *c*(*f*), |*H*(*f* + *f_c_*)|^2^ = *h*(*f*), and *P_nn_*(*f*) = *n*(*f*). The SCNR difference is defined as:

(32) $$\begin{array}{l} \Delta \text{SCNR}={\text{SCNR}}_{\text{max}}-{\text{SCNR}}_{\text{pm}}\\ \;\;\;\;\;\;\;\;\;\;\;\;\;\;\;\;\;\;\;=\int_{-B/2}^{B/2}\frac{h(f)n(f)({ɛ}_{opt}(f)-{ɛ}_{\mathrm{pm}}(f))}{\left(n(f)+c(f){ɛ}_{opt}(f))(n(f)+c(f){ɛ}_{\mathrm{pm}}(f)\right)}df \end{array}$$

Then, we have that:

(33) $$\Delta SCNR\leq \int_{-B/2}^{B/2}\frac{h(f)}{n(f)}|{ɛ}_{\mathrm{pm}}(f)-{ɛ}_{opt}(f)|df$$

Applying the Cauchy–Schwarz inequality to equation (33), we have that:

(34) $$\begin{array}{l} {\left(\Delta \mathrm{SCNR}\right)}^{2}\leq {\left[\int_{-B/2}^{B/2}\frac{h(f)}{n(f)}|{ɛ}_{\mathrm{pm}}(f)-{ɛ}_{\mathrm{opt}}(f)|df\right]}^{2}\\ \;\;\;\;\;\;\;\;\;\;\;\;\;\;\;\;\;\;\;\;\;\;\;\leq \int_{-B/2}^{B/2}{\left(\frac{h(f)}{n(f)}\right)}^{2}df\cdot \int_{-B/2}^{B/2}{\left({ɛ}_{\mathrm{pm}}(f)-{ɛ}_{\mathrm{opt}}(f)\right)}^{2}df \end{array}$$

Simply, it is given by:

(35) $${\left(\Delta \mathrm{SCNR}\right)}^{2}\leq {\int_{-B/2}^{B/2}\left(\frac{|H(f+f_{c}){|}^{2}}{P_{nn}(f)}\right)}^{2}df\cdot \int_{-B/2}^{B/2}{\left({ɛ}_{\mathrm{pm}}(f)-{ɛ}_{\mathrm{opt}}(f)\right)}^{2}df$$

We let:

(36) $${\int_{-B/2}^{B/2}\left(\frac{|H(f+f_{c}){|}^{2}}{P_{nn}(f)}\right)}^{2}df={\tilde{\text{K}}}^{\prime }$$

According to the previous assumptions in Section 2, the spectrum of extended target impulse response *H*(*f* + *f_c_*) and the noise PSD *P_nn_*(*f*) are both supposed to be deterministic and to be known, K̃′ can be viewed as a constant, we have:

(37) $${\left(\Delta \mathrm{SCNR}\right)}^{2}\leq {\tilde{\text{K}}}^{\prime }\cdot \int_{-B/2}^{B/2}{\left({ɛ}_{\mathrm{pm}}(f)-{ɛ}_{\mathrm{opt}}(f)\right)}^{2}df$$

## Appendix B

From the definition of ESD difference *G*(*ϕ*) in (24), the partial derivative of *G*(*ϕ*) with respect to *ϕ_k_* which indicates the phase of *u_pm_*(*t*) at the *k_th_* sampling time, is:

(38) $$\begin{array}{l} \frac{\partial G(\phi )}{\partial \phi k}=j2c^{4}T_{s}^{4}F_{s}\{e^{j2\phi k}\sum_{m=0}^{M-1}{\left(ϒ(f_{m})\right)}^{2}+e^{j\phi k}\sum_{m=0}^{M-1}\Phi (f_{m})\\ \;\;\;\;\;\;\;\;\;\;\;\;\;\;\;\;\;\;\;\;\;-e^{-j\phi k}\sum_{m=0}^{M-1}\Phi *(f_{m})-e^{-j2\phi k}\sum_{m=0}^{M-1}{\left(ϒ*(f_{m})\right)}^{2}\} \end{array}$$

where *M* = *B*/*F_s_*, *f_m_* = −*B*/2 + *mF_s_*, $c=\sqrt{E/T}$, (·)* is the conjugate operator, *F_s_* is the sampling interval in frequency domain, *T* is the signal duration time, *T_s_* is the sampling interval, *E* is the transmit energy, and ϒ(*f_m_*) and Φ(*f_m_*) are defined by:

(39) $$ϒ(f_{m})=e^{-j2\pi f_{m}kT_{s}}\sum_{n=0,n\neq k}^{N-1}e^{-j(\phi_{n}-2\pi f_{m}nT_{s})}$$

(40) $$\Phi (f_{m})=\left({\left|ϒ(f_{m})\right|}^{2}-\frac{|U_{opt}(f_{m}){|}^{2}}{c^{2}T_{s}^{2}}+1\right)ϒ(f_{m})$$

where *N* = *T*/*T_s_*. Moreover, the second-order partial derivative is:

(41) $$\begin{array}{l} \frac{\partial^{2}G(\phi )}{\partial \phi_{k}^{2}}=-2c^{4}T_{s}^{4}F_{s}\{e^{j2\phi k}\sum_{m=0}^{M-1}2{\left(ϒ(f_{m})\right)}^{2}+e^{j\phi k}\sum_{m=0}^{M-1}\Phi (f_{m})\\ \;\;\;\;\;\;\;\;\;\;\;\;\;\;\;\;\;\;\;\;\;\;\;\;+e^{-j\phi k}\sum_{m=0}^{M-1}\Phi *(f_{m})+e^{-j2\phi k}\sum_{m=0}^{M-1}2{\left(ϒ*(f_{m})\right)}^{2}\} \end{array}$$

Therefore, the appropriate phase element *ϕ_k_* that minimizes *G*(*ϕ*) must satisfy *∂G*(*ϕ*)/*∂ϕ_k_* = 0 and $\partial^{2}G(\phi )/\partial \phi_{k}^{2}>0$. We let $\text{A}=\sum_{m=0}^{M-1}{\left(ϒ(f_{m})\right)}^{2}$, $\text{B}=\sum_{m=0}^{M-1}\Phi (f_{m})$ and *x* = *e*^*jϕ*_*k*_^, so the first derivative is:

(42) $$\frac{\partial G(\phi )}{\partial \phi k}=j2c^{4}T_{s}^{4}F_{s}(Ax^{2}+Bx-B*x^{-1}-A*x^{-2})$$

and the second derivative is:

(43) $$\frac{\partial^{2}G(\phi )}{\partial \phi_{k}^{2}}=-2c^{4}T_{s}^{4}F_{s}(Ax^{2}+Bx+B*x^{-1}+A*x^{-2})$$

It is clear that for *∂G*(*ϕ*)/*∂ϕ_k_* = 0, we must have:

(44) $$\text{A}x^{4}+\text{B}x^{3}+\text{ B}*x-\text{A}*=0$$

Because *G*(*ϕ*) is real and periodic with the period equal to 2π, the extreme points are present in pairs within the analysis interval. Thus, the solutions of Equation (44) must exit and be conjugated in the analysis interval. Besides, the coefficients of Equation (44) that are conjugated in pairs make the solutions be unity amplitude and be easily solved by a general quartic equation solving method. Furthermore, we must obtain the appropriate solution *x_o_*, which can make:

(45) $$\text{A}x_{o}^{2}+\text{B}x_{o}+\text{ B}*x_{o}^{-1}+\text{ A}*x_{o}^{-2}<0)$$

be held. In other words, the appropriate phase *ϕ_k_*, which is the angle of *x_o_*, can minimize the ESD difference *G*(*ϕ*) defined in Equation (24). It should be noted that the objective function in this optimization problem is non-convex, and therefore the solution is guaranteed to be only locally optimal.
